# Hydroxyl Radical-Induced Oxidation on the Properties of Cathepsin H and Its Influence in Myofibrillar Proteins Degradation of *Coregonus peled In Vitro*

**DOI:** 10.3390/foods13162531

**Published:** 2024-08-14

**Authors:** Xuemei Fan, Mengjie Ma, Pingping Liu, Xiaorong Deng, Jian Zhang

**Affiliations:** 1 School of Food Science and Technology, Shihezi University, Shihezi 832003, China; m15288425245@163.com (X.F.); 20202011004@stu.shzu.edu.cn (M.M.); liupp0222@163.com (P.L.); dxr20099@163.com (X.D.); 2 Key Laboratory of Agricultural Product Processing and Quality Control of Specialty (Co-Construction by Ministry and Province), School of Food Science and Technology, Shihezi University, Shihezi 832003, China; 3 Key Laboratory for Food Nutrition and Safety Control of Xinjiang Production and Construction Corps, School of Food Science and Technology, Shihezi University, Shihezi 832003, China

**Keywords:** cathepsin H, oxidation, protein degradation, *Coregonus peled*

## Abstract

The most frequently occurring protein modification in fish postmortem is oxidization, which further affects meat quality through multiple biochemical pathways. To investigate how hydroxyl radicals affect the structure of cathepsin H and its ability to break down myofibrillar proteins in *Coregonus peled*, cathepsin H was oxidized with 0, 0.1, 0.5, 1, 5, and 10 mM H_2_O_2_ and subsequently incubated with isolated myofibrillar proteins. The results showed that as the H_2_O_2_ concentration increased, the carbonyl and sulfhydryl contents of cathepsin H significantly increased and decreased, respectively. There were noticeable changes in the α-helix structures and a gradual reduction in UV absorbance and fluorescence intensity, indicating that oxidation can induce the cross-linking and aggregation of cathepsin H. These structural changes further reduced the activity of cathepsin H, reaching its lowest at 10 mM H_2_O_2_, which was 53.63% of the activity at 0 mM H_2_O_2_. Moreover, desmin and troponin-T all degraded at faster rates when cathepsin H and myofibrillar proteins were oxidized concurrently as opposed to when cathepsin H was oxidized alone. These findings provide vital insights into the interaction mechanism between oxidation, cathepsin H, as well as myofibrillar protein degradation, laying a groundwork for understanding the molecular mechanisms underlying changes in fish meat quality after slaughter and during processing.

## 1. Introduction

*Coregonus peled* is a characteristic coldwater fish in Xinjiang, which is gaining popularity among consumers worldwide due to its high nutritional value [1,2]. However, the quality of *Coregonus peled* deteriorates during post-slaughter storage mainly due to fish softening [3,4]. Fish texture is recognized as a key indicator of its freshness, affecting consumer taste and satisfaction [5,6]. Due to its high moisture content, active endogenous enzymes, and susceptibility to biochemical and microbial effects, the texture of fish meat typically changes and the flavor deteriorates faster than other types of meat, such as lamb, beef, and pork, which in turn reduces the food and commercial value of fish [7,8,9]. Among them, the degradation of myofibrillar proteins by cathepsins is recognized as one of the critical factors causing tissue softening in fish [10,11]. Numerous studies have shown that cathepsin B, D, H, and L have significantly increased activity in post-slaughter muscle and are crucial in the degradation of myofibrillar proteins and tissue softening in fish after slaughter [12,13]. For example, cathepsin B degrades myosin heavy chain and troponin, while cathepsin L and H specifically degrade actin and troponin-T [12,14]. Studies on fish or beef muscle cathepsin H have been limited compared to cathepsin B, D, and L, which may be due to the low levels of this enzyme in postmortem muscle [12,15,16]. However, in mammals, cathepsin H has been proven to degrade myofibrillar proteins, and its degradation is faster than that of cathepsin B in animal skeletal muscle [17,18]. Similarly, it has been found that cathepsin H in Atlantic halibut is highly related to protein content changes and plays a vital role in juice loss [14]. Another study also found a highly positive correlation between cathepsin B, D, and H activities and increased MFI in postmortem grass carp, and these cathepsins play an important role in postmortem softening and structural changes in grass carp [19]. Thus, although the activity of cathepsin H is lower in postmortem muscle, its vital role in fish softening cannot be ignored, and further investigation is required to fully understand its contribution to the degradation of fish myofibrillar proteins in fish.

It is widely known that the production and storage of fish are often accompanied by the occurrence of oxidation, which is considered one of the most significant variables influencing the quality of fish meat [4]. Oxidation during fish storage mainly consists of fat oxidation and protein oxidation, the former has been widely studied and proved to be an important factor affecting the color and flavor of meat during post-slaughter storage [16]. It has been found that proteins in muscle tissue, especially myofibrillar proteins, are as susceptible to reactive oxygen species (ROS) as fat [20]. In recent years, researchers have been paying close attention to protein oxidation in the field of meat science. Protein oxidation is carried out by free radicals such as hydroxyl radicals, superoxide radicals, and hydrogen peroxide, which can directly catalyze protein oxidation reactions [21,22]. Among them, hydroxyl radicals are recognized as the major oxidizing radicals affecting the biochemical properties of myofibrillar proteins in vitro [20]. In addition, hydroxyl radical oxidation systems (Fe^3+^, ascorbic acid and different concentrations of H_2_O_2_) have been widely used in the study of protein oxidation in food systems [20,23]. Numerous studies have found that hydroxyl radicals (·OH) alter the structure of myofibrillar proteins and increase their sensitivity to protein hydrolysis, which in turn accelerates the degradation of myofibrillar proteins [4,24,25]. Many studies also reported that the oxidative modification of μ-calpain by hydroxyl radicals (·OH) alters its enzymatic activity, which in turn affects the ability to degrade myofibrillar proteins [3,26,27,28]. However, the research focus has been on the oxidation of myofibrillar proteins and the oxidation of μ-calpain, respectively. Some scholars found that hydroxyl radical-induced oxidation led to a decrease in cathepsin D, B and L enzyme activity [2,29]. It was tentatively deduced that oxidation also alters fish texture by altering cathepsin activity. Hence, the study of cathepsin oxidation is important to establish the link between oxidation and fish softening. In actual transport and processing, both cathepsins and myofibrillar proteins may be affected by oxidation at the same time. However, there are fewer studies on cathepsin oxidation, especially on which of the cathepsin H and myofibrillar proteins is more susceptible to oxidation, how cathepsin H oxidizes, and how both oxidation processes affect the breakdown of myofibrillar proteins.

In order to exclude the interference of other enzymes and reaction factors, in the present study, an in vitro model was established by an ex vivo test to oxidize active cathepsin H and isolated myofibrillar proteins, and the effect of cathepsin H oxidation on the degradation of myofibrillar proteins in *Coregonus peled* was studied by combining electrophoresis (SDS-PAGE) and Western blotting. The aim was to assess how different oxidation levels affect the ability of cathepsin H to degrade myofibrillar proteins using in vitro models, providing a theoretical basis for inhibiting the activity of cathepsin H, appropriately controlling protein oxidation, and maximizing the maintenance of the quality of *Coregonus peled* during storage and processing, as well as providing an experimental basis for revealing the mechanism of the influence of protein oxidation for the fish meat’s softness.

## 2. Materials and Methods

### 2.1. Sample Preparation

Approximately 20 fresh *Coregonus peled* (average bodyweight 1000 ± 50 g) were obtained from an industrial fisheries production facility (SaiHu Fishery Technology Development Co., Ltd., Bole, China). They were delivered to the lab via cold-chain transportation in approximately 5 h. The dorsal white muscle was sliced into portions that weighed approximately 10 g each, quickly frozen with liquid nitrogen, and then immediately stored in a refrigerator at −80 °C until needed.

### 2.2. Preparation of Myofibrillar Proteins (MPs)

MPs were extracted in the published way by Lu et al. [1] with several alterations. Briefly, 4 g of fish mince from *Coregonus peled* was homogenized with 40 mL of pre-cooled deionized water at a homogenization speed of 10,000 rpm (Scientz Co., Ningbo, China) for 60 s. After homogenization, the sediment was centrifuged at 8000× *g* for 10 min at 4 °C using a GL21M centrifuge (Hunan Kaida Scientific Instrument Co., Ltd., Changsha, China). The precipitate was then redissolved with 0.3% NaCl solution and centrifuged again. The precipitate was dissolved by adding 40 mL of buffer (20 mM Tris-HCl, 600 mM NaCl, pH 7.0) and diluted with pre-chilled ultrapure water at a ratio of 1:4 (*v*/*v*) and centrifuged again under the same conditions as above. Finally, the precipitate was dissolved in 0.6 M NaCl solution and filtered through a three-layer sieve, and the filtration was myofibrillar protein. The protein concentration of MPs was measured using the BCA Protein Assay Kit and then adjusted to 10 mg/mL.

### 2.3. Oxidative Treatment of Cathepsin H and Myofibrillar Protein

The oxidation system was established using the previously published procedure by Park et al. [30] with a few minor adjustments. The active cathepsin H and isolated MPs (10 mg/mL) were, respectively, incubated with Fenton’s reagent at 4 °C for 60 min. Fenton’s reagent was a 50 mM phosphate buffer (pH 6.0) containing 0.01 mM FeCl_3_, 0.1 mM ascorbic acid, and varying concentrations of H_2_O_2_, which were 0, 0.1, 0.5, 1, 5, and 10 mM, respectively. At the end of incubation, the reaction was terminated by adding 1 mM EDTA. Subsequently, the mixture was washed twice with 4-fold pre-cooled diluted water and centrifuged at 8000× *g* for 5 min at 4 °C. The final precipitates were cathepsin H and MPs without the Fenton reagent.

### 2.4. Measurement of the Carbonyls in Cathepsin H

With slight modifications, the previously described approach was used to measure the carbonyl content [31]. Two 70 µL oxidized cathepsin H solutions were reacted with 1 mL of 10 mM DNPH (diluted in 2 M hydrochloric acid) and 2 M hydrochloric acid solution (as a blank control), respectively, for 60 min at room temperature in the dark. At the end of the reaction, 1 mL of 20% trichloroacetic acid (*w*/*v*) was then added and centrifuged (8000× *g*, 5 min, 4 °C). The precipitate was collected, and 1 mL of ethanol–ethyl acetate mixture (1:1, *v*/*v*) was then added to wash the precipitate three times and centrifuged under the same conditions. The precipitate was then mixed with 3 mL of 20 mM phosphate buffer solution (pH 6.0) containing 6 M guanidine hydrochloride, centrifuged again, and the precipitate was removed. Absorbance was measured at 370 nm, and the carbonyl content was calculated using the molar absorption coefficient (22,000 M^−1^CM^−1^) and expressed as mmol/mg protein.

### 2.5. Determination of the Sulfhydryl Content in Cathepsin H

The measurement of the total sulfhydryl content was based on the former description with the appropriate version [3]. First, 1 mL of oxidized cathepsin H suspension was mixed with 4 mL of phosphate buffer (0.1 M, 8 M urea, 3% SDS, pH 7.0), which was followed by the addition of 1 mL of 10 mM 2-nitrobenzoic acid dissolved in 0.1 M phosphate buffer (pH 7.0), and the reaction was performed in the dark for 15 min at room temperature. The absorbance of the sample was measured at 412 nm. The total sulfhydryl content of the protein sample was then calculated by dividing the absorbance value by the molar extinction coefficient of sulfhydryl groups (13,600 M^−1^CM^−1^). The results were expressed as nanomoles per milligram of protein (nmol/mg protein).

### 2.6. Structural Analysis of Cathepsin H

#### 2.6.1. Endogenous Fluorescence Spectrum

Fluorescence spectra were obtained using a modification of the approach employed by Jiang et al. [32]. Oxidized cathepsin H was diluted to 0.1 mg/mL. The spectra of oxidized cathepsin H were recorded using a fluorescence spectrophotometer at wavelengths from 300 to 400 nm with an excitation wavelength of 283 nm.

#### 2.6.2. UV Absorption Spectrum

The UV absorption spectra were performed according to the method previously reported by Qiu et al. [33] with some minor modifications. Oxidized cathepsin H was diluted to 0.1 mg/mL. Then, the UV absorption spectra at 230–500 nm wavelengths of cathepsin H with different oxidized concentration treatments were recorded using a UV spectrophotometer.

#### 2.6.3. Circular Dichroism (CD) Spectral

Changes in the secondary structure of cathepsin H were detected with the MOS-450 circular dichroism spectrometer (Biological, Rennes, France) as a previously described method with just minor modifications [34]. The protein concentration was 0.2 mg/mL. A quartz cuvette with an optical path length of 1.0 mm was used and scanned in the far-ultraviolet band (190–240 nm). The resolution, scanning speed, and response values of the spectrometer were set to 0.5 nm, 60 nm/min, 0.25 s, and 1 s, respectively.

### 2.7. Assay of Calponin H Activity

The determination of cathepsin H activity was mainly based on the method of Seymour et al. [35] with small modifications. The reaction system consisted of 98 µL of 0.1% Brij35, 40 µL of 0.4 M phosphate buffer (pH 6.8) containing 5 mM EDTA, 20 µL of 10 mM cysteine, and 60 µL of oxidized cathepsin H solution. The mixture was placed in a water bath at 40 °C for 2 min, and then 40 µL of substrate was added to continue the water bath for 30 min. Subsequently, 300 µL of termination solution (0.1 M sodium acetate and 0.1 M sodium chloroacetate, pH 4.3) was added to stop the reaction. The measurement of fluorescence intensity was obtained using the spectrophotometer (970 CRT, Shanghai Yidian Analytical Instruments Co., Ltd., Shanghai, China) at the excitation wavelength (370 nm) and emission wavelength (460 nm). Cathepsin H activity was expressed relative to the activity at the 0 mM H_2_O_2_ sample.

### 2.8. Incubations

To assess how much protein oxidation affects cathepsin H’s rate of MPs degradation, oxidized and untreated active cathepsin H was incubated with known concentrations of MPs [36]. A 1 mL of MPs dilution was added to 65 µL of oxidized or untreated cathepsin H and incubated for 120 min at 25 °C. At the end of the incubation, the reaction was terminated by adding 500 µL of 0.1 M EDTA. The samples were then mixed thoroughly by adding 2× Sampling Buffer (125 mmol/L tris, 4% SDS, 20% glycine, 10% β-mercaptoethanol, 0.01% bromophenol blue) and heated at 95 °C for 5 min to ensure proper denaturation. Finally, the samples were stored at −80 °C until analysis.

### 2.9. SDS-PAGE and Western Blotting

Methods were referred to as previously described [37] with minor modifications. The 15% separator gel was used for the detection of troponin-T, desmin and myosin heavy chain (MHC) with 10 µg of sample loaded per well. While 12% of the separating gel was used for detection of actin, loading 7 µg of sample per well. All samples were 5% concentrated gel. The gels were run on a Bio-Rad Mini-Protean II system (Bio-Rad Laboratories, Hercules, CA, USA) at a constant voltage of 80 V. After electrophoresis, Coomassie brilliant blue R-250 was used to stain MHC in 15% gels. Troponin T and desmin from 15% gels and actin from 12% gels were transferred to PVDF membranes (Millipore, Bedford, MA, USA) using a semi-dry transmembrane apparatus (Bio-Rad Laboratories, Hercules, CA, USA) at a constant current of 200 mA. The membranes were closed with TBST solution containing 5% skimmed milk (20 mM Tris, 150 mM NaCl, 0.05% Tween 20, pH 7.5) for 120 min at room temperature. At the end of the closure, the PVDF membranes were cleaned using TBST solution four times for 15 min each time. Troponin-T and desmin were then incubated with mouse monoclonal antibodies (ab10214, Abcam, Cambridge, UK and ab8470, Abcam, Cambridge, UK) diluted at 1:4000, respectively, at 4 °C overnight. Actin was incubated with mouse monoclonal antibody (3E9, Sigma, Louis, MO, USA) diluted at 1:2000 at 4 °C overnight. At the end of the incubation, the membrane was cleaned again under the same conditions as above. Subsequently, the membranes were incubated with a secondary antibody solution (ab6789, Abcam, Cambridge, UK) diluted at 1:15,000 for 60 min at room temperature. Finally, the membrane was washed again. Drops of ECL chemiluminescent reagent were added to the PVDF membrane and scanned using a gel imaging system (Bio-Rad Laboratories, Hercules, CA, USA), and the density of the target bands was analyzed using Quantity One.

### 2.10. Statistical Analysis

All experiments in this experiment were repeated three times, and the data were expressed as mean ± standard deviation (SD). Data were assessed by variance (ANOVA) and the Duncan multiple range test with SPSS 26.0. Differences were regarded as statistically significant at *p* < 0.05.

## 3. Results and Discussion

### 3.1. Protein Oxidation of Cathepsin H

#### 3.1.1. Carbonyl Content

**Figure 1:**
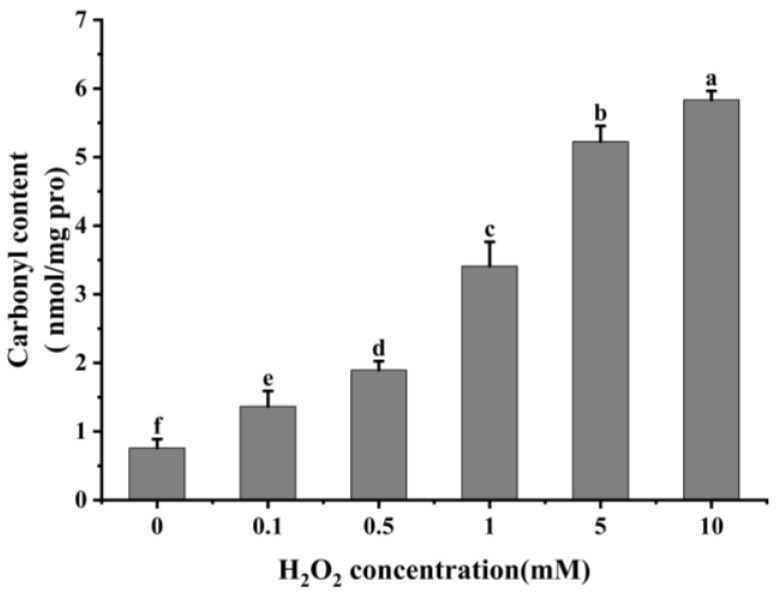
Effect of different concentrations of H_2_O_2_ on carbonyl content in cathepsin H. Different letters indicate the significance of changes (*p* < 0.05).

It is usual practice to evaluate the degree of protein oxidation using the carbonyl content of proteins [21,38]. As shown in Figure 1, cathepsin H’s carbonyl content rose remarkably (*p* < 0.05) with increasing H_2_O_2_ concentration after oxidation at 4 °C for 60 min. The carbonyl content of cathepsin H was 0.76 nmol/mg at 0 mM H_2_O_2_ treatment, whereas it increased to 5.83 nmol/mg at 10 mM H_2_O_2_ oxidation. This result follows the same trend found by previous studies which found that the carbonyl content in μ-calpain increased significantly with increasing H_2_O_2_ concentration [3,27]. The increase in carbonyl content is due to the reaction of Fe^2+^ with H_2_O_2_ in the Fenton reaction system to produce hydroxyl radicals, and the amino acid residues in the side chain of proteins are susceptible to the hydroxyl radicals, resulting in the formation of carbonyl derivatives [39,40]. Additionally, the formation of carbonyl derivatives may also be closely related to oxidation-induced peptide bond breakage [21].

#### 3.1.2. Total Sulfhydryl

**Figure 2:**
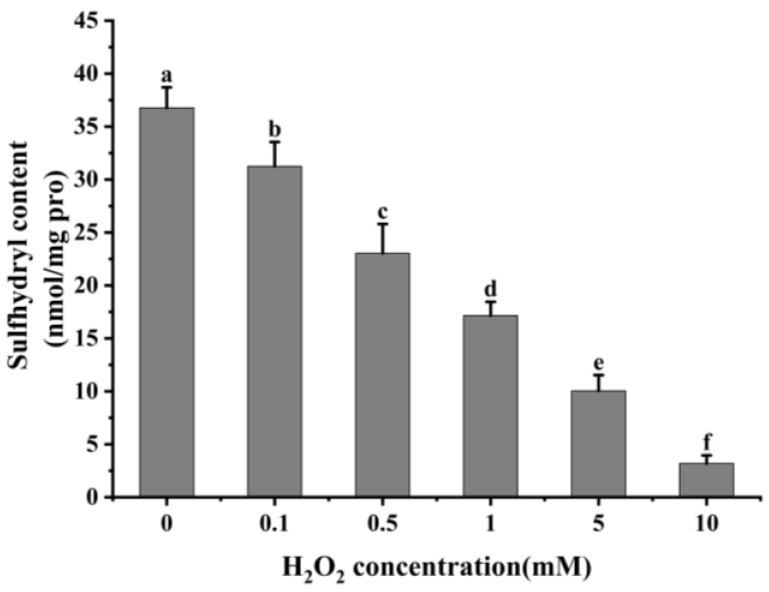
Effect of different concentrations of H_2_O_2_ on total sulfhydryl content in cathepsin H. Different letters indicate the significance of changes (*p* < 0.05).

**Figure 3:**
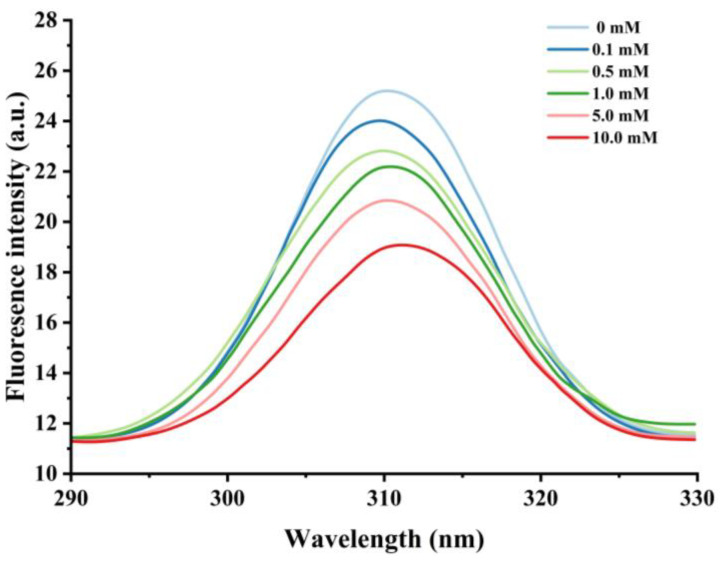
Effect of various H_2_O_2_ concentrations on the intronic fluorescence intensity in cathepsin H.

**Figure 4:**
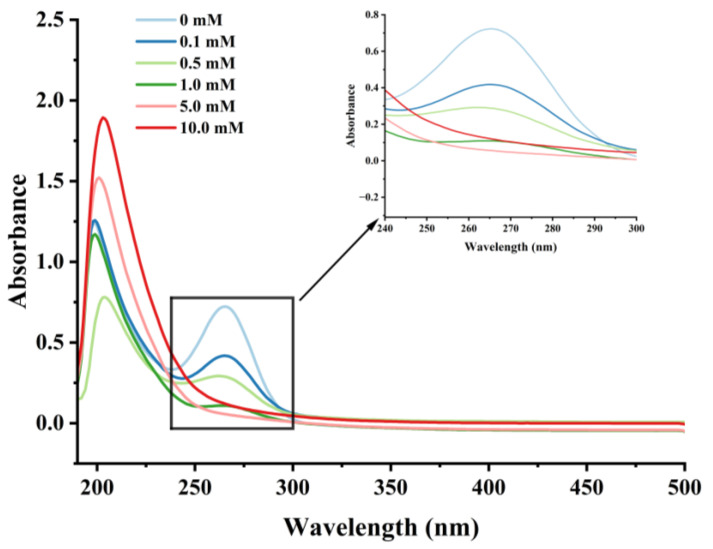
Effect of various H_2_O_2_ concentrations on the UV absorption spectrum in cathepsin H.

**Figure 5:**
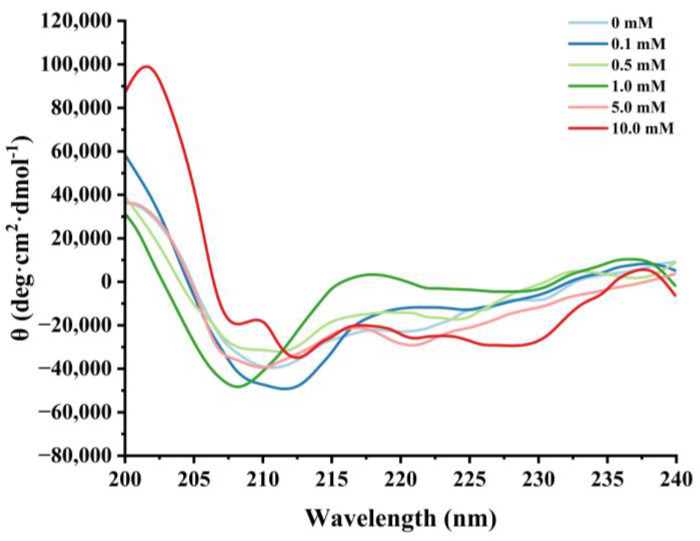
Effect of various H_2_O_2_ concentrations on the circular dichroism in cathepsin H.

Reactive oxygen species readily oxidize protein cysteine residues, and the level of sulfhydryl groups is often used as a reflection of the degree of protein oxidation [41,42]. As shown in Figure 2, as the concentration of H_2_O_2_ increased, the total sulfhydryl content in cathepsin H reduced dramatically (*p* < 0.05). The total sulfhydryl content of cathepsin H was 36.76 nmol/mg at 0 mM H_2_O_2_. This content was reduced to a minimum of 3.19 nmol/mg at 10 mM H_2_O_2_. Previous studies have similarly reported a significant decrease in total sulfhydryl content with increasing H_2_O_2_ concentration [42,43]. Zhang et al. [4] also found that the total sulfhydryl content decreased from 78.29 to 63.74 nmol/mg when the concentration of H_2_O_2_ reached 10 mmol/L. The decrease in total sulfhydryl content indicates that cathepsin H unfolds in response to increasing concentrations of H_2_O_2_, releasing free sulfhydryl groups, which are susceptible to oxidation to form intramolecular or intermolecular disulfide bonds [4]. In addition, free sulfhydryl groups generate sulfinic and sulfonic acids during oxidation, resulting in the formation of protein aggregates [22]. In contrast to the notable increase in carbonyl content, the substantial reduction in sulfhydryl content further enhanced the impact of oxidation on the structure of cathepsin H. Thus, these results indicate that the amino acid residues in cathepsin H can be attacked by hydroxyl radicals, leading to alterations in its tertiary and secondary structure (Figure 3, Figure 4 and Figure 5), suggesting that the experiment successfully modeled the state of cathepsin H under six different oxidation levels.

### 3.2. Impact of In Vitro Oxidation on Cathepsin H Structure

#### 3.2.1. Changes in Intrinsic Fluorescence

Nonpolar amino acid residues such as tryptophan, tyrosine, and phenylalanine are sensitive to changes in protein polarity or microenvironment, and fluorescence measurements of these groups are commonly used to monitor changes in proteins’ tertiary structure [44]. When the excitation wavelength is above 290 nm, the emission spectrum of tryptophan can be obtained by avoiding the interference of other amino acids [45], and thus the excitation wavelength of 290–330 nm was used to further assess the degree of oxidation of cathepsin H by hydroxyl radicals. As shown in Figure 3, the unoxidized cathepsin H has a broad band of maximum fluorescence intensity at 310 nm. The fluorescence intensity decreased significantly with increasing H_2_O_2_ concentration, from 25.22 at 0 mM H_2_O_2_ to 19.11 at 10 mM, which is a decrease of about 24.23%. Meanwhile, a slight redshift in λ_max_ (i.e., a 1 nm increase in wavelength) was observed in the 10 mM H_2_O_2_ treatment. The results suggest that hydroxyl radical oxidation leads to the unfolding of the cathepsin H structure, and the tryptophan residues embedded inside the hydrophobic cavity of the protein are gradually exposed to the polar environment, which causes the fluorescence intensity to decrease and the maximum emission wavelength to redshift [42,46]. In addition, the formation of protein aggregates may have obscured certain nonpolar amino acid residues, which could account for the decline in endogenous fluorescence intensity [47]. Yang et al. [10] reached a similar conclusion in their study on the conformational changes of myofibrillar fibers affected by the hydrolysis of endogenous proteins under acidic conditions. Thus, the decrease in endogenous fluorescence intensity confirms that the cathepsin H tertiary structure is altered.

#### 3.2.2. Changes in UV Absorption Spectral

UV spectroscopy can detect changes in chromophores, such as the side chains of aromatic amino acids, reflecting to some extent the conformational changes of proteins [44,48]. Cathepsin H’s UV spectra at various H_2_O_2_ concentrations are displayed in Figure 4. In the UV-spectroscopic analysis, a solvent absorption peak was observed at approximately 210 nm, which is a characteristic feature arising from the solvent’s own absorption of UV light. Meanwhile, the highest absorption peak was observed near 265 nm for cathepsin H, representing changes in aromatic amino acids such as threonine, tyrosine, and phenylalanine [42], which gradually weakened with increasing H_2_O_2_ concentration. In addition, the UV absorption peaks of cathepsin H were diminished to almost no peaks at higher concentrations of H_2_O_2_. The results indicated that the aromatic amino acid residues on the surface of cathepsin H were subjected to oxidative modification, resulting in an aggregation of cathepsin H during oxidation, forming high molecular weight polymers that covered up some nonpolar amino acid residues [47]. This is consistent with previous reports that the maximum UV absorption peak of myofibrillar fibrillar proteins diminishes with increasing concentrations of H_2_O_2_ [34,42]. Zhang et al. [49]. also indicated that tyrosine is susceptible to oxidation, and the coupling of two tyrosine residues can further proceed through covalent bonds to form dityrosine, which enhances protein cross-linking either intramolecularly or intermolecularly. Furthermore, a slight redshift in the UV spectrum was observed at 0.1, 0.5, and 1 mM H_2_O_2_, moving from 265 to 267 to 268 nm, further demonstrating that hydroxyl radical oxidation induced conformational changes in cathepsin H [50]. The above results also confirmed that hydroxyl radical oxidation leads to the alteration of cathepsin H’s tertiary structure.

#### 3.2.3. Changes in Circular Dichroism

Circular dichroism is a routine method for assessing the secondary structure of proteins [25,51]. Negative peaks near 208 nm and 220 nm were observed in the CD spectra of natural cathepsin H, which indicates the presence of a major α-helical structure in cathepsin H (Figure 5). Satoh et al. [52] suggested that the negative peaks near 208 nm and 222 nm in circular dichroism can be used to evaluate the changes in the α-helical content of the protein. Furthermore, the intensity of these characteristic peaks of cathepsin H began to flatten out with increasing H_2_O_2_ concentration, and the negative peaks weakened at 0.5 mM H_2_O_2_. Hydrogen bonding between the amide C=O and N-H groups is the main force that stabilizes the a-helix conformation [49]. Oxidative reactions can lead to protein unfolding or peptide bond breaking, resulting in a reduction in hydrogen bonding [39,48]. The results suggest that the oxidation process might cause the rearrangement of hydrogen bonds within cathepsin H, affecting the stability of the α-helix and thus leading to the alteration of cathepsin H’s secondary structure [53].

### 3.3. Effects of In Vitro Oxidation on Cathepsin H Activity

**Figure 6:**
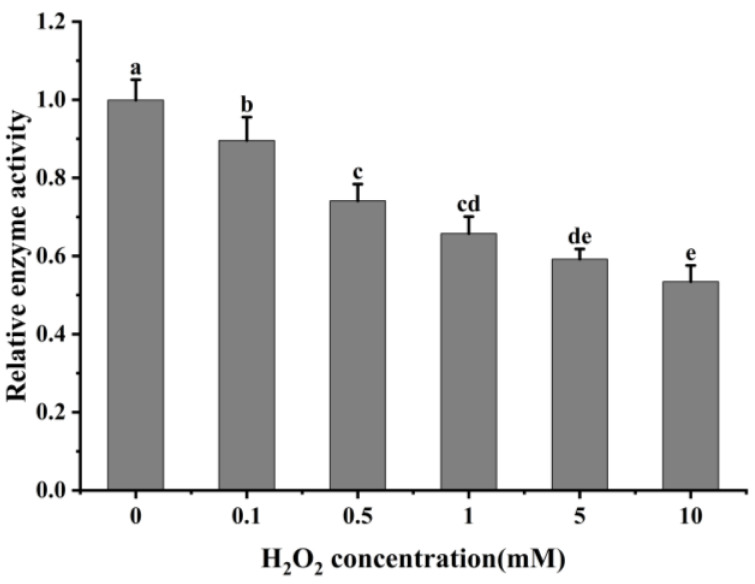
Impact of varying H_2_O_2_ on cathepsin H activity. Different letters indicate the significance of changes (*p* < 0.05).

The oxidative modification of cathepsin H by hydroxyl radicals results in a change in its conformation, and to determine whether this process affects its protease activity, the activity of cathepsin H was measured. As shown in Figure 6, the relative activity of cathepsin H gradually decreased with increasing H_2_O_2_ concentration with a significant decrease at 0–0.5 mM (*p* < 0.05). When exposed to 10 mM H_2_O_2_, its activity dropped to only 53.63% of that at 0 mM H_2_O_2_. The result indicates that the oxidative action of hydroxyl radicals significantly inhibits the activity of cathepsin H, and this inhibitory effect intensifies with increasing H_2_O_2_ concentration. This decrease in activity is likely due to cathepsin H being a class of cysteine protease with cysteine activation and substrate cleavage sites, and oxidation by H_2_O_2_ alters the cysteine active sites [3,54]. This result follows the trend found in previous studies [26,28,54], which found a gradual decrease in caspase-3 or μ-calpain activity at lower concentrations of H_2_O_2_, while at higher concentrations, H_2_O_2_ led to a rapid inactivation of caspase-3 or μ-calpain. Similarly, another study also reported that cathepsin D, B and L decreased its activity after oxidative treatment with H_2_O_2_ [2,29]. However, Liu et al. [27] reported that μ-calpain activity showed a first increase and then a decrease with increasing H_2_O_2_ concentration. Qin et al. [3]. also revealed that μ-calpain activity increased with increasing hydrogen peroxide concentration. This difference can be explained by the differences in the sensitivity of different proteases to H_2_O_2_. The above results indicate that oxidative treatment alters the spatial structure of cathepsin H, resulting in an aggregation of the enzyme protein molecules and hiding certain amino acids and active sites, thus reducing its activity. This analysis is consistent with results from ultraviolet and intrinsic fluorescence spectroscopy.

### 3.4. In Vitro Degradation of Myofibrillar Proteins by Oxidized Cathepsin H

#### 3.4.1. General

**Figure 7:**
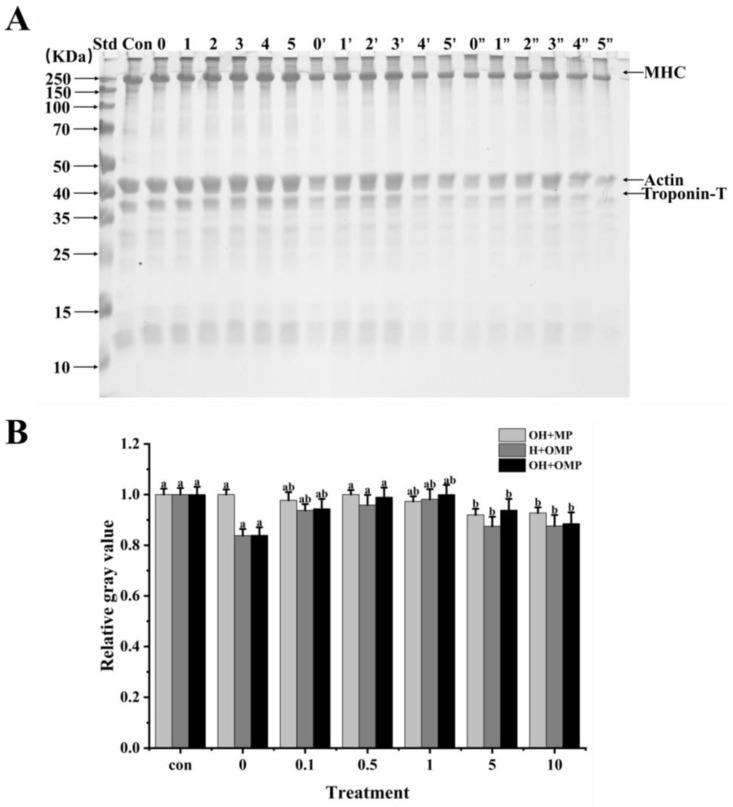
Electrophoretic analysis of the degradation of myofibrillar proteins by cathepsin H at different oxidation levels (**A**) and relative gray values of intact MHC bands (**B**). Con represents control (without cathepsin H); 0, 1, 2, 3, 4, and 5 represent incubation groups at 0, 0.1, 0.5, 1, 5, and 10 mM H_2_O_2_-oxidized cathepsin H and unoxidized MPs (OH + MP); 0′, 1′, 2′, 3′, 4′ and 5′ represent incubation groups at 0, 0.1, 0.5, 1, 5, 10 mM H_2_O_2_-oxidized MPs and unoxidized cathepsin H (H + OMP); 0″, 1″, 2″, 3″, 4″ and 5″ represent incubation groups at 0, 0.1, 0.5, 1, 5, and 10 mM H_2_O_2_-oxidized cathepsin H and oxidized MPs (OH + OMP). Different letters indicate the significance of changes (*p* < 0.05).

In order to ascertain how oxidation affects cathepsin H’s capacity for breaking down MPs in *Coregonus peled*, MPs (oxidized or unoxidized) was incubated independently of cathepsin H (oxidized or unoxidized). Changes in the degradation of MPs were then analyzed using SDS-PAGE by comparing samples in which cathepsin H was oxidized alone (OH + MP), MPs were oxidized alone (H + OMP), and both were oxidized simultaneously (OH + OMP) (Figure 7). It can be clearly observed in Figure 7 that cathepsin H degrades MHC, actin and troponin-T differently, indicating that hydroxyl radical oxidation affected cathepsin H’s capacity to hydrolyze MPs. The magnitude and pattern of degradation of these proteins were further determined using protein immunoblot analysis.

#### 3.4.2. Myosin Heavy Chains

Myosin heavy chain (MHC) plays a crucial role in skeletal muscle function and is the main component of thick filaments in muscle segments [55]. It has been shown that MHC is the main target of protease attack and its degradation process directly affects muscle texture and softness [56]. As shown in Figure 7A,B, MHC exhibited different degrees of degradation under different oxidative treatment conditions. The MHC band intensity among the OH + MP sample did not change significantly, and the cumulative bands could be observed at the top of the gel, progressively increasing at 0–1 mM H_2_O_2_ and then becoming lighter at 5–10 mM (Figure 7A). Meanwhile, the degradation of MHC did not change much at 0–1 mM H_2_O_2_ concentration, while MHC was slightly degraded at the H_2_O_2_ concentration exceeding 1 mM (Figure 7B). The results indicated that the oxidative modification of cathepsin H inhibited its degradation of MHC, which may be related to the oxidative inhibition of cathepsin H activity (Figure 6). The same phenomenon was also observed by Liu et al. [27], where highly oxidized µ-calpain activity was inhibited, further reducing its degradation of MHC. In contrast, Qin et al. [3] reported that oxidized µ-calpain could more easily break down MHC and showed that this was due to an increase in µ-calpain activity as a result of the oxidation.

When MPs were oxidized but cathepsin H was not oxidized (H + OMP), the MHC bands became thicker and then thinner, and there was a slight accumulation of bands at the top of the gel (Figure 7A). At the same time as the MPs’ oxidation level rose, the degradation of the MHC decreased and then increased with the lowest MHC degradation at 1 mM H_2_O_2_ (Figure 7B). This may be due to the fact that under oxidative conditions, disulfide bonds or other chemical bonds can lead to the cross-linking of MPs and the formation of polymers, which inhibit the action of cathepsin H to a certain extent, leading to a decrease in MHC degradation [44,48]. Some researchers have suggested that oxidation increases MHC degradation by promoting the cross-linking and aggregation of MPs, altering its chemical and physical binding sites, and increasing its susceptibility to proteases [27,36]. This is consistent with studies showing that higher levels of MPs oxidation contribute to µ-calpain and caspase-3 to gradually degrade MHC [57]. In addition, the trends of MHC band intensity and degradation after incubation with oxidized MPs and oxidized cathepsin H (OH + OMP) were similar to those of the H + OMP treatment (Figure 7A,B). However, the degree of MHC degradation in the OH + OMP treatment was higher than in the OH + MP treatment at 0, 0.1, and 0.5 mM H_2_O_2_ but slightly lower at 1 and 5 mM H_2_O_2_, and it was slightly lower than in the H + OMP treatment at 0.5, 1, and 5 mM H_2_O_2_. The results indicated that at lower oxidation levels, cathepsin H oxidized alone degraded MHC at a lower rate than MPs oxidized alone and both simultaneously.

#### 3.4.3. Actin

**Figure 8:**
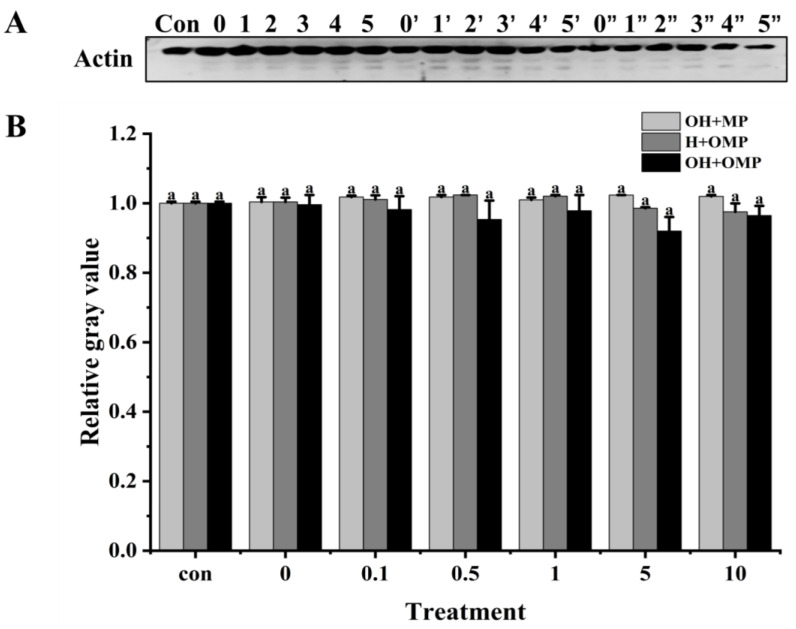
Typical immunostaining (**A**) and relative gray values analysis (**B**) of actin in cathepsin H-treated myofibrillar fibrillar proteins at different oxidation levels. Con represents control (without cathepsin H); 0, 1, 2, 3, 4, and 5 represent incubation groups at 0, 0.1, 0.5, 1, 5, and 10 mM H_2_O_2_-oxidized cathepsin H and unoxidized MPs (OH + MP); 0′, 1′, 2′, 3′, 4′ and 5′ represent incubation groups at 0, 0.1, 0.5, 1, 5, 10 mM H_2_O_2_-oxidized MPs and unoxidized cathepsin H (H + OMP); 0″, 1″, 2″, 3″, 4″ and 5″ represent incubation groups at 0, 0.1, 0.5, 1, 5, and 10 mM H_2_O_2_-oxidized cathepsin H and oxidized MPs (OH + OMP). Different letters indicate the significance of changes (*p* < 0.05).

Actin (~42 kDa) is the major protein in myofiber filaments, and maintaining the structural integrity of actin filaments plays an important role in the toughness of fish muscle [55]. Actin has been reported to be specifically hydrolyzed by cathepsins in post-slaughter muscle, which is detrimental to fish texture [12]. As presented in Figure 8A, it was clearly observed that the actin bands and their degradation fragments were thicker in the OH + MP group and H + OMP group, while they became lighter in the OH + OMP group. The actin degradation did not change significantly in the OH + MP group despite the increasing oxidation levels of cathepsin H (*p* > 0.05), suggesting that oxidized cathepsin H has a lower capacity to hydrolyze actin (Figure 8B). It was possible that the oxidative modification resulted in a change in the spatial conformation of cathepsin H, masking the active site and thus affecting the substrate specificity and efficiency of the enzyme, coinciding with the change in its activity change (Figure 6). Similarly, actin remained unchanged at lower levels of oxidation of MPs in the H + OMP group, whereas a slight degradation of actin occurred when the H_2_O_2_ concentration exceeded 1 mM (Figure 8B). But compared with the OH + MP group, the degradation of H + OMP actin was 3.7% and 4.4% higher at 5 and 10 mM H_2_O_2_, respectively, suggesting that the moderate oxidation of MPs may protect actin, whereas higher oxidation promotes its degradation. This may be caused by the oxidation of MPs leading to the aggregation of protein interactions or an alteration of their sensitivity to proteases [2]. Xue et al. [36] similarly reported no significant difference in actin degradation when oxidized MPs in beef were incubated with active μ-calpain, whereas Smuder et al. [31] found that the degradation of rat actin by μ-calpain and caspase-3 increased with increasing levels of oxidized MPs. The degradation of actin may vary depending on the species, the type of protease, and the treatment conditions.

Moreover, at 0.1–10 mM H_2_O_2_, the degradation of actin was 3.5%, 6.3%, 3.1%, 10.1%, and 5.4% higher in the OH + OMP group than in the OH + MP group, and it increased by 2.8%, 6.8%, 4.1%, 6.7%, and 1.1%, respectively, compared to that in the H + OMP group, but this change was not significant (*p* > 0.05; Figure 8B). The results showed that neither cathepsin H oxidation alone nor the simultaneous oxidation of MPs and cathepsin H had a remarkable effect on actin degradation. Some researchers have suggested that actin is quite stable and less susceptible to the external environment except under high oxidation and resistant to protease attack [58]. These discoveries reveal the complex effects of oxidation on the degradation process of actin, holding significant implications for understanding the mechanisms behind fish muscle softening.

#### 3.4.4. Desmin

**Figure 9:**
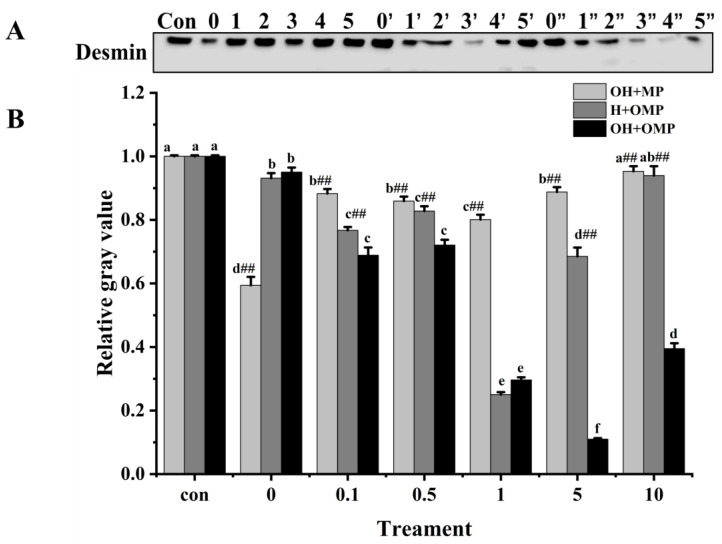
Typical immunostaining (**A**) and relative gray values analysis (**B**) of desmin in cathepsin H-treated myofibrillar fibrillar proteins at different oxidation levels. Con represents control (without cathepsin H); 0, 1, 2, 3, 4, and 5 represent incubation groups at 0, 0.1, 0.5, 1, 5, and 10 mM H_2_O_2_-oxidized cathepsin H and unoxidized MPs (OH + MP); 0′, 1′, 2′, 3′, 4′ and 5′ represent incubation groups at 0, 0.1, 0.5, 1, 5, 10 mM H_2_O_2_-oxidized MPs and unoxidized cathepsin H (H + OMP); 0″, 1″, 2″, 3″, 4″ and 5″ represent incubation groups at 0, 0.1, 0.5, 1, 5, and 10 mM H_2_O_2_-oxidized cathepsin H and oxidized MPs (OH + OMP). Different letters are used to indicate statistically significant (*p* < 0.05) differences within groups across experimental conditions; “##” indicates a highly significant difference (*p* < 0.01) between the OH + MP group or the H + OMP group and the OH + OMP group.

Desmin (~55 kDa) is a principal intermediate filament protein connecting adjacent myofibrils and is essential in maintaining muscle structure and organization integrity [59]. Desmin appears to be very sensitive to postmortem proteolysis [44]. The magnitude of desmin degradation was measured after the incubation of MPs with cathepsin H to determine whether cathepsin H is involved in desmin degradation. As depicted in Figure 9A, the degradation pattern of desmin stands out distinctly from those of MHC and actin. At 0 mM H_2_O_2_, the degradation of desmin in the OH + MP group was the most significant, which was likely due to the lack of oxidation of cathepsin H, preserving its enzymatic activity (Figure 9B). Compared with the control group, the degradation of desmin in the OH + MP group first rose and then sharply declined at 0.1–10 mM H_2_O_2_ (*p* < 0.05; Figure 9B). Notably, at 1 mM H_2_O_2_, the degradation reached a peak of 19.9%, indicating that desmin is sensitive to cathepsin H and that moderate oxidation enhances its hydrolytic activity of desmin. However, the excessive oxidation of cathepsin H at higher H_2_O_2_ concentrations reduces its hydrolytic capacity. This result is in agreement with previous findings in which they found that the incubation of unoxidized myofibrillar proteins with oxidized μ-calpain revealed a significant increase in the degradation of interstitial thread proteins at H_2_O_2_ below 0.5 mM, whereas the reverse was true at H_2_O_2_ above 0.5 mM [27]. Interestingly, the change in desmin degradation in the H + OMP group was similar to that of the OH + MP group with a maximum degradation of 74.9% at 1 mM H_2_O_2_. The results suggested that the moderate oxidation of MPs enhances the degradation of desmin by cathepsin H, whereas high oxidation inhibits its degradation. This may be caused by the fact that the high oxidation of MPs altered the sensitivity of desmin to degradation by cathepsin H and protected it from hydrolysis. Zhang et al. [4] showed similar results: when MPs are oxidized in vitro, desmin becomes less susceptible to μ-calpain breakdown. Chen et al. [24] also reported that the in vitro oxidation of desmin promoted the degradation of caspase-3 and caspase-6 while impeding the breakdown of μ-calpain.

Additionally, in the OH + OMP group, desmin degradation increased significantly in the 0–5 mM H_2_O_2_ (0.5 mM excluded) with an increase of 70.4% for 5 mM H_2_O_2_ (*p* < 0.05; Figure 9B). At 0.1–10 mM H_2_O_2_, desmin degradation was all highly significantly higher in the OH + OMP group than in the OH + MP group, by 21.9%, 16.1%, 63.0%, 87.6%, and 58.6%, respectively (*p* < 0.01). Similarly, it was highly significantly higher (*p* < 0.01) at 0.1–10 mM H_2_O_2_ (except 1 mM) than that of the H + OMP group (10.2%, 12.9%, 83.9.1%, and 57%, respectively). In addition, desmin degradation was higher in the H + OMP group than in the OH + MP group at 0.1–10 mM H_2_O_2_, especially at 0.1, 1 and 5 mM H_2_O_2_. But previous studies have observed that desmin degradation in simultaneous oxidation by protease and MPs is lower than in oxidation by protease alone, which may be the inconsistent sensitivity of different proteases to hydroxyl radical oxidation [2,27]. These results suggested that the simultaneous oxidation of cathepsin H and MPs enhances desmin degradation, whereas the oxidation of cathepsin H alone inhibits desmin degradation, which is possibly because oxidation alters the structure of both the protease and the substrate and promotes the formation of new or more active degradation sites.

#### 3.4.5. Troponin-T

**Figure 10:**
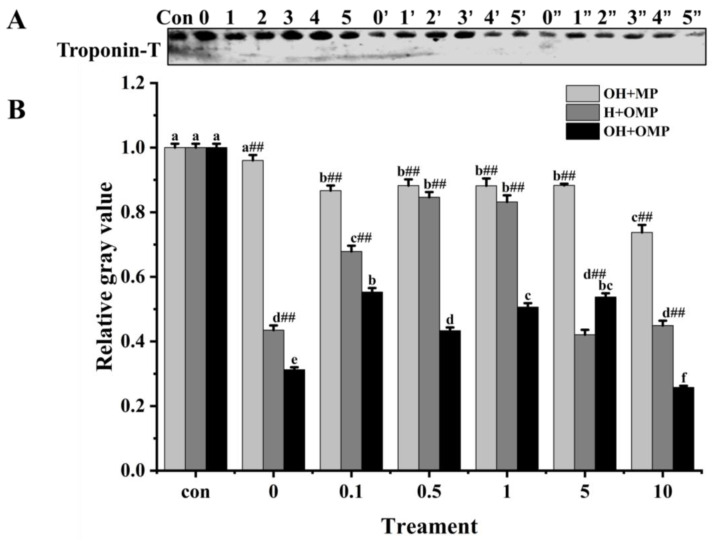
Typical immunostaining (**A**) and relative gray values analysis (**B**) of troponin-T in cathepsin H-treated myofibrillar fibrillar proteins at different oxidation levels. Con represents control (without cathepsin H); 0, 1, 2, 3, 4, and 5 represent incubation groups at 0, 0.1, 0.5, 1, 5, and 10 mM H_2_O_2_-oxidized cathepsin H and unoxidized MPs (OH + MP); 0′, 1′, 2′, 3′, 4′ and 5′ represent incubation groups at 0, 0.1, 0.5, 1, 5, 10 mM H_2_O_2_-oxidized MPs and unoxidized cathepsin H (H + OMP); 0″, 1″, 2″, 3″, 4″ and 5″ represent incubation groups at 0, 0.1, 0.5, 1, 5, and 10 mM H_2_O_2_-oxidized cathepsin H and oxidized MPs (OH + OMP). Different letters are used to indicate statistically significant (*p* < 0.05) differences within groups across experimental conditions; “##” indicates a highly significant difference (*p* < 0.01) between the OH + MP group or the H + OMP group and the OH + OMP group.

Troponin-T is the component of the troponin complex that binds to myosin and is generally considered to be the most sensitive subunit of the troponin molecule to protein hydrolysis [60]. Moreover, the breakdown of troponin-T in fish muscle post-slaughter, which is induced by cathepsin, is considered crucial in the softening of the fish flesh structure [12]. Troponin-T bands were thickest in the OH + MP group and thinnest in the OH + OMP group (Figure 10A). The degradation of troponin-T in the OH + MP group gradually increased with the increase in the degree of oxidation of cathepsin H with no significant difference between 0.1 mM and 5 mM H_2_O_2_ (*p* > 0.05), whereas it was significantly increased by 23.3% at 10 mM H_2_O_2_, probably owing to the severe disruption of the structure of highly oxidized cathepsin H exposing more active sites. The results are inconsistent with reports that the oxidation of μ-calpain increased the degradation of troponin-T, which may be due to different effects of oxidation on protease activity [3].

However, in the H + OMP group, troponin-T degradation decreased significantly and subsequently increased (*p* < 0.05) as MPs oxidation increased with the lowest degradation at 0.5 and 1 mM H_2_O_2_, reaching 15.4% and 16.8%. This change was in line with the MHC degradation pattern (Figure 7B). The results showed that the moderate oxidation of MPs inhibited the hydrolysis of troponin-T by cathepsin H, while high oxidation promoted its hydrolysis of troponin-T. It is possible that a moderate oxidation-induced MPs aggregation increases structural hindrance and decreases their sensitivity by proteases, whereas high oxidation severely destroys the MPs’ structure [4].

Furthermore, troponin-T degradation exhibited a marked increase in the OH + OMP sample (*p* < 0.05; Figure 10B) and was more pronounced compared to the H + OMP group (except for 5 mM H_2_O_2_) and the OH + MP group (*p* < 0.01). However, this result is slightly different from previous studies, which found that the simultaneous oxidation of proteases and MPs prevented troponin-T from degrading, possibly as a result of different sensitivities of different proteases to oxidation [2,27]. Similarly, at concentrations of 0.1, 5, and 10 mM H_2_O_2_, the H + OMP group demonstrated a greater degradation of troponin-T by 21.7%, 52.3%, and 39.1%, respectively, compared to the OH + MP group. These results were in accordance with the desmin changes (Figure 9B). The above results suggested that troponin-T degradation is slow when cathepsin H is oxidized alone, but the simultaneous oxidation of tissue cathepsin H and MPs promotes the hydrolysis of troponin-T, which is probably due to reasons similar to those of desmin.

## 4. Conclusions

The present results showed that the oxidative modification of cathepsin H by hydroxyl radicals resulted in a remarkable rise in the carbonyl content and a significant drop in the total sulfhydryl content of cathepsin H (*p* < 0.05). Meanwhile, cathepsin H underwent structural alterations that resulted in a gradual loss of enzyme activity, which in turn delayed the degradation of desmin and troponin-T, and at a lower level of oxidation, it also delayed the degradation of MHC, while it had no significant effect on the actin degradation. Furthermore, the oxidation of MPs played a large role in MPs’ degradation by oxidized cathepsin H. Desmin and troponin-T were more readily degraded by oxidized cathepsin H after the incubation of both oxidized cathepsin H and oxidized MPs, suggesting that the simultaneous oxidation of MPs and cathepsin H together contributed to the softening of the texture of the fish meat. This research not only profoundly uncovers the mechanisms of protein oxidation in complex muscle systems but also provides a solid theoretical foundation for effectively controlling protein oxidation in *Coregonus peled* fish meat during storage and processing. However, fish protein oxidation involves complex physiological and biochemical processes, and further studies are needed to investigate the degradation of MPs by cathepsin H under different conditions, and other cytoskeletal proteins need to be considered in future studies, aiming for a comprehensive understanding of how protein oxidation impacts the quality of fish meat.

## Data Availability

The data presented in this study are available on request from the corresponding author. The data are not publicly available due to privacy restrictions.
